# Targeting the post-synaptic proteome has therapeutic potential for psychosis in Alzheimer Disease

**DOI:** 10.1038/s42003-023-04961-5

**Published:** 2023-06-02

**Authors:** J. M. Krivinko, M. R. DeChellis-Marks, L. Zeng, P. Fan, O. L. Lopez, Y. Ding, L. Wang, J. Kofler, M. L. MacDonald, R. A. Sweet

**Affiliations:** 1 Department of Psychiatry, University of Pittsburgh School of Medicine, Pittsburgh, PA USA; 2 Department of Neurobiology, University of Pittsburgh School of Medicine, Pittsburgh, PA USA; 3 Department of Biostatistics, University of Pittsburgh School of Public Health, Pittsburgh, PA USA; 4 Department of Pharmaceutical Sciences, Computational Chemical Genomics Screening Center, University of Pittsburgh School of Pharmacy, Pittsburgh, PA USA; 5 Department of Neurology, University of Pittsburgh School of Medicine, Pittsburgh, PA USA; 6 Department of Pathology, University of Pittsburgh School of Medicine, Pittsburgh, PA USA

**Keywords:** Alzheimer's disease, Psychosis, Translational research

## Abstract

Individuals with Alzheimer Disease who develop psychotic symptoms (AD + P) experience more rapid cognitive decline and have reduced indices of synaptic integrity relative to those without psychosis (AD-P). We sought to determine whether the postsynaptic density (PSD) proteome is altered in AD + P relative to AD-P, analyzing PSDs from dorsolateral prefrontal cortex of AD + P, AD-P, and a reference group of cognitively normal elderly subjects. The PSD proteome of AD + P showed a global shift towards lower levels of all proteins relative to AD-P, enriched for kinases, proteins regulating Rho GTPases, and other regulators of the actin cytoskeleton. We computationally identified potential novel therapies predicted to reverse the PSD protein signature of AD + P. Five days of administration of one of these drugs, the C-C Motif Chemokine Receptor 5 inhibitor, maraviroc, led to a net reversal of the PSD protein signature in adult mice, nominating it as a novel potential treatment for AD + P.

## Abstract (web summary)

The postsynaptic density proteome is found to be altered specifically in brain tissue from Alzheimer’s Disease patients that experience psychosis and is thence computationally shown to be a potential therapeutic target.

## Introduction

Psychotic symptoms affect ~40–60% of individuals with Alzheimer Disease (AD)^1^. AD subjects with psychosis (AD + P) experience greater cognitive dysfunction early in disease progression prior to psychosis onset^2,3^ and more rapid cognitive decline compared to AD subjects without psychosis (AD-P)^1–4^. Current treatments for psychosis in AD with antipsychotics have limited efficacy, do not mitigate the more rapid cognitive decline, and confer excess mortality^5^. In addition, AD + P is associated with worse outcomes than AD-P, including higher rates of aggression^6^, caregiver distress^7^, functional decline^8^, institutionalization^9^, and mortality^10^. Thus, there is strong motivation to identify the biology underlying psychosis in AD in hopes of developing a targeted, more efficacious intervention.

The risk for psychosis in AD is to a large extent genetically determined, with an estimated heritability of 60%, indicative of a distinct biologic vulnerability^4,11,12^. Because it has long been recognized that synapse loss is the strongest neuropathologic correlate of cognitive decline in AD^13,14^, it has been hypothesized that the vulnerability to AD + P arises from greater synaptic impairment in AD + P than AD-P. Prior studies that compared AD + P to AD-P subjects on a number of correlates of synapse integrity, including gray matter volumes, cerebral glucose utilization or blood flow, or gray matter concentrations of the membrane breakdown products, glycerophosphoethanolamine and glycerophosphocholine, have found support for this hypothesis across neocortical, but not medial temporal regions (reviewed in ref. ^15^). Most recently, we examined gray matter levels of a limited panel of 190 synaptic proteins in individuals with AD + P and found reductions in canonical postsynaptic density (PSD) proteins relative to AD-P subjects^16^. These differences exceeded those which could be accounted for by any differences in neuropathology burden between the groups^16^, or by reductions in the corresponding mRNA transcripts due to greater excitatory neuron loss in AD + P^17^.

None of these prior studies have directly examined the PSD in AD + P. We therefore conducted a proteomic analysis of PSD fractions from dorsolateral prefrontal cortex of a large group of individuals with AD + P and AD- P. To facilitate interpretation of differences between AD + P and AD-P we also included a reference group of cognitively normal elderly subjects. We found a global shift to reduced levels of PSD proteins in AD + P subjects relative to both AD-P and cognitively normal subjects. There was a global shift towards lower levels in PSDs from AD + P relative to AD-P subjects, including lower levels of a network of kinases, proteins regulating Rho GTPases, and proteins regulating the actin cytoskeleton. Using computational systems pharmacology we identified several currently approved drugs predicted to reverse the PSD protein level differences between AD + P and AD-P. Five days of exposure to one of these drugs, maraviroc, led to a net reversal of the PSD protein signature in adult mice, nominating it as a novel potential treatment for AD + P.

## Results

### PSD abundance in AD with and without psychosis

Prior to evaluating the full cohort, we validated our PSD enrichment in a subset of the AD cases (Fig. S1). To explore whether psychosis status globally affected the PSD yield in AD, we first evaluated the association of AD + P with total gray matter yield of PSD protein (Fig. S2). Mean µg PSD/µg of gray matter protein was significantly reduced in AD + P relative to both AD-P and elderly cognitively normal subjects. The reduction in PSD yield in AD-P relative to elderly cognitively normal subjects was not significant.

### PSD protein levels in AD with and without psychosis

**Fig. 1:**
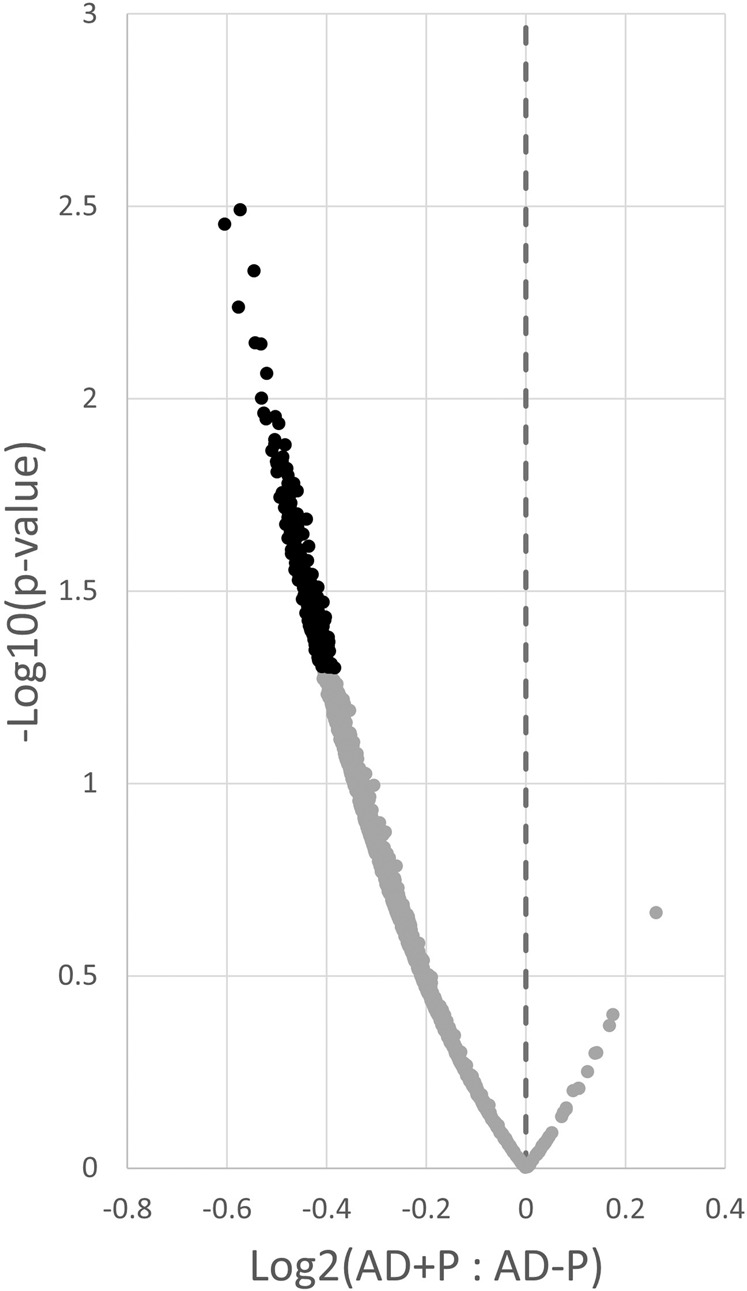
Distribution of levels of 1,613 PSD proteins quantified in DLPFC using peptides present in 100% of AD + P and AD-P subjects. Distributions of log_2_ ratios are shown for all proteins, adjusted for covariates, Age, PMI, Sex, APOE*E4, Lewy Body presence, and phospho-Tau area fraction. The dashed vertical line represents no difference in the ratio of protein levels between groups. Black points indicate the 240 proteins with nominally significant differences in levels in AD + P relative to AD-P (*p* < 0.05). AD + P is characterized by a significant shift towards lower PSD protein levels compared to AD-P.

We next evaluated yield-adjusted PSD protein levels, conservatively limiting our initial analysis to the peptides present in 100% of subjects, comprising 1,613 proteins (Fig. 1). There was a significant shift in abundance of almost all proteins to lower levels in AD + P compared to AD-P (t = −90.6, df = 1612, *p* < 2 × 10^−307^). We conducted additional analyses to confirm the robustness of this global shift. The global reduction was similarly present in the larger set of PSD proteins quantified using peptides present in ≥50% of samples (Fig. S3a). Similarly, the global reduction of PSD protein levels persisted when covarying for excitatory neuron proportion, a measure we previously reported is reduced in AD + P relative to AD-P^17^ (Fig. S3b, c).

**Fig. 2:**
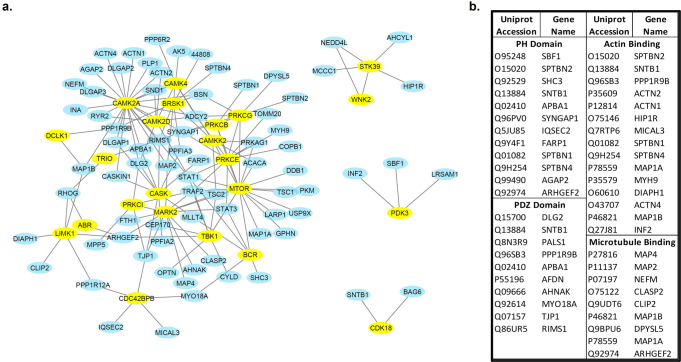
The interaction network of kinases and proteins nominally differentially expressed (*p* < 0.05) in PSD of AD + P relative to AD-P. To explore the impact of AD + P on the phosphorylation network, we identified 23 kinases in the in the functional annotation analysis of the 240 differentially expressed proteins. We then used String v11.5^58^ to search the interactions between these 23 kinases with the remaining 217 nominally differentially expressed proteins. We found that 76/217 (35.0%) proteins were reported to interact with the 23 kinase proteins. As a comparison, only 348/1373 (25.2%) of the non-differentially expressed proteins (defined by AD + P vs AD-P *p* > 0.05) were reported to interact with the 23 kinases (χ^2^ = 8.97, df = 1, *p* = 0.0027). **a** Shows the interactions of the kinases with the 76 proteins. Yellow symbols=kinases, Blue symbols=other proteins. **b** Largest functional groups from among the 76 interacting proteins.

No individual protein level was significantly reduced with an adjusted *p* < 0.05 (Supplementary Data 1). Thus, to characterize the nature of the broader signal of global reduction, we examined the 240 most reduced proteins (those with unadjusted *p* < 0.05). Functional annotation analysis of these 240 PSD proteins relative to a background of the 1,613 proteins that were quantified in 100% of subjects revealed strong enrichment for regulators of the actin cytoskeleton, a critical determinant of post-synaptic dendritic spine structure and function (Supplementary Data 2). These included actin binding proteins and regulators of Rho GTPase signaling such as Pleckstrin homology (PH) domain containing Rho guanine nucleotide exchange factors (RhoGEFs) and Rho GTPase activating proteins (RhoGAPs). Additionally, there was enrichment for 23 protein serine/threonine kinases (Supplementary Data 2). These 23 kinases, which are important regulators of RhoGEFs, RhoGAPs, and actin binding proteins^18^, interact within a network that was itself enriched for the remaining 217 PSD proteins with nominally significant reductions in AD + P (Fig. 2). In addition to kinases, PH domain containing proteins, and actin binding proteins, the network included PDZ domain containing proteins and microtubule binding proteins, suggestive of disrupted signaling to additional post-synaptic structural elements (Fig. 2b). Choice of a more liberal threshold for peptide inclusion (e.g., present call ≥50%) did not substantially alter the functional annotation findings, although now enrichment for histone proteins also emerged (Supplementary Data 3).

### Comparison of AD + P and AD-P with elderly cognitively normal subjects

While our focus was on the comparison of AD + P relative to AD-P, our dataset provided an opportunity to examine how the PSD is altered in these groups relative to our reference group of elderly cognitively normal subjects. Both AD groups were characterized by a significant shift towards lower PSD protein levels compared to cognitively normal reference group, with greater reductions in AD + P than in AD-P (Figure S4a–c). As a result, 836/1613 (51.8%) proteins had an adjusted p < 0.05 in AD + P in comparison to the reference group, whereas the corresponding number was 2/1613 (0.001%) proteins for the AD-P group (Supplementary Data 4). The protein whose abundance was most altered between the AD and comparison subjects was APP, due solely to the increased levels of two peptides found within the Aβ sequence (RHDSGYEVHHQK and KLVFFAEDVGSNK, Supplementary Data 4 and noted as Aβ in Fig. S4c). Overall, the protein alterations present in the two AD groups relative to elderly cognitively normal subjects were highly correlated (Fig. S4c). As a consequence, both groups showed enrichment of differentially expressed proteins involved in protein translation and of glycoproteins. Nevertheless, functional annotation of the proteins that differentiated AD + P or AD-P from reference subjects revealed some differential patterns of enrichment. PSD proteins that differentiated AD + P from elderly cognitively normal subjects included enrichment for proteins involved in calcium signaling, synaptic function, and cytoskeletal proteins. PSD proteins that differentiated AD-P from elderly cognitively normal subjects were enriched for proteins involved in protein quality control, ion channels, and small GTPases (Supplementary Data 5).

We also undertook to contrast the PSD signature of AD±P relative to elderly cognitively normal subjects to the most extensive prior examination of the functional enrichment of proteins in total gray matter homogenates of individuals with AD and elderly controls subjects, reported in^19^. This contrast is summarized in Fig. S5. The differentially expressed proteins in the PSDs of both AD + P and AD-P shared with the overall gray matter homogenate enrichment for ribosomal proteins and glycoproteins. Other gray matter functional enrichments shared with either AD + P (Synapse, Mitochondria) or AD-P (Cell-ECM interactions, Ubiquitination) may indicate that some of the overall signal detected in AD homogenate derives primarily from subgroups of individuals with or without psychosis. Finally, enrichments detected only in the PSD preparations (e.g. impacting Calcium signaling, ion transport, and the cytoskeleton) may reflect pathologic cascades that are most prominent within PSDs and not as readily detected in the multicellular, multicompartmental, gray matter homogenate. As such, these functions may represent postsynaptic-specific targets for intervention for cognitive impairment more generally.

### Relationship to genetic risk for AD with psychosis

We recently reported genome-wide gene-based tests of association with psychosis in AD^11^. A total of 239 of the 240 PSD proteins that were nominally significantly reduced in AD + P had available corresponding gene-based tests of association (Supplementary Data 6, and Supplemental Materials). Of these 239 genes whose proteins were assayed, 11 genes which were nominally altered at the protein level in AD + P also exhibited at least nominal evidence of genetic association with AD + P relative to AD-P. Notable among these were the actin binding protein genes *SYNPO*, *SYNE1*, *ALDOA*, and *VPS16*. Also included were the kinase *PRKCI*, and the GAP *TBC1D10B*.

### Predicted drug activity against the AD + P PSD protein network

We next sought to identify existing pharmacotherapies with potential abilities to target the disrupted PSD signature of AD + P. We identified 50 gene knockout datasets in which the resulting changes to the transcriptome were correlated with the PSD protein signature of AD + P (Supplementary Data 7). We identified 46 drugs targeting these genes, 7 of which showed the desired effect (inhibitor/antagonist or positive regulator) on the targeted gene (Table 1). To further assess, computationally, the potential post-treatment effect of the nominated drugs on PSD protein levels in AD + P, two additional analyses were performed. First, the expression signatures due to knockdown of the upstream-genes were correlated with only the 240 PSD proteins nominally significantly reduced in AD + P to ensure that the correlation between the knockdown signature and AD + P persisted when examining the most dysregulated proteins. This analysis identified that *MTOR* had a correlation with the top 240 proteins that were opposite (negative) to its overall correlation with the 1613 proteins, excluding it, and its positive regulator, pimecrolimus, from further consideration.

**Table 1:** Drugs with predicted beneficial effects.

Drug	Indication	Targets gene	Drug-target Action	Correlation Between Gene Knockdown Signature and AD + P PSD Protein Signature
Fostamatinib	Chronic immune thrombocytopenia	*AURKB*	Inhibitor	Negative
Maraviroc	CCR5-tropic HIV-1 infection	*CCR5*	Antagonist	Negative
Leronlimab	Investigated for the treatment of a number of cancers and HIV	*CCR5*	Antagonist	Negative
Ibalizumab	HIV-1	*CCR5*	Antagonist	Negative
Procaine	Local anesthetic primarily in oral surgery	*DNMT1*	Inhibitor	Negative
Epigallocatechin Gallate	Investigated for the treatment of Hypertension and Diabetic Nephropathy.	*DNMT1*	Inhibitor	Negative
Pimecrolimus	Mild to moderate atopic dermatitis	*MTOR*	Potentiator	Positive*

*MTOR positively correlated with the entire AD + P PSD proteome signature (*N* = 1613 proteins), but unlike the other target genes demonstrated an opposing pattern of correlations with the top differentially expressed PSD proteins (*N* = 240).

Second, we undertook to validate the gene signatures of the remaining candidate drugs. Of these, leronlimab, ibalizumab, and epigallocatechin gallate were not found in the LINCS database for evaluation of their induced transcriptome expression signatures, and procaine was not tested in CNS cells. Two of the remaining three medications, fostamatinib and maraviroc demonstrated transcriptome expression signatures that were negatively correlated with the AD + P PSD protein signature (Signed Jaccard Indexes of −0.0025 and −0.0009, respectively, Supplementary Data 8).

### Effect of maraviroc on the PSD proteome

**Fig. 3:**
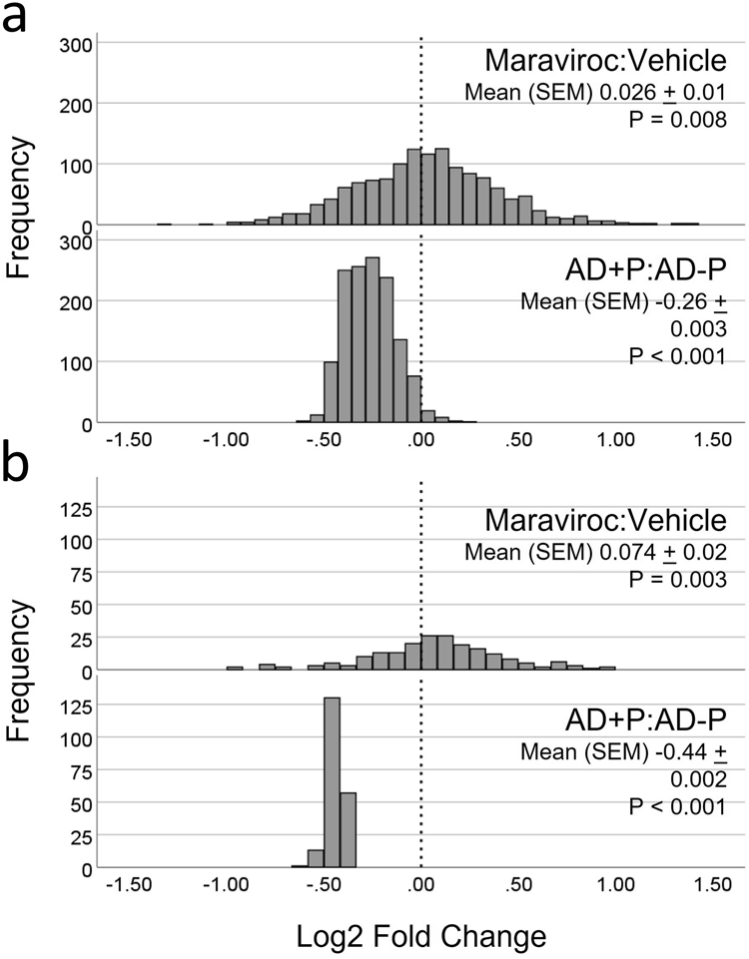
Maraviroc effects on the PSD proteome signature of AD + P. Adult C57Bl/6 J WT mice were randomized to daily intraperitoneal injection of maraviroc 50 mg/kg or vehicle for 5 days. 1370 of the 1613 proteins that comprised our PSD signature of AD + P, including 201 of the 240 PSD proteins that were nominally significantly reduced in AD + P, were successfully quantified. **a** Maraviroc treatment had a net effect of increasing the abundance of 1370 proteins. The corresponding fold changes in AD + P vs AD-P for the identical proteins are also shown. **b** Maraviroc treatment similarly had a net effect of increasing abundance in the subset of 201 proteins. *P* values are from one sample t-tests where the null was a Log2(fold-change)=0.

We looked to confirm our prediction for one of the above drugs, maraviroc, by testing its effects on the PSD proteome in mice. We administered either 50 mg/kg maraviroc dissolved in a vehicle solution of 18% ethanol in normal saline or an equivalent volume of vehicle solution alone to three-month-old C57Bl/6 J WT mice for 5 days. We successfully quantified 1370 of the 1613 proteins that comprised our PSD signature of AD + P, including 201 of the 240 PSD proteins that were nominally significantly reduced in AD + P. Maraviroc treatment had a net effect of increasing PSD protein abundance, with a more marked effect in the subset of 201 proteins most disrupted in AD + P (Fig. 3, Supplementary Data 9). The Signed Jaccard Index was −0.01186 for maraviroc’s effect on the AD + P PSD proteome signature.

## Discussion

We hypothesized that AD + P arises from a more severe synaptopathy than that which is present in AD-P, based on multiple studies that have compared indirect measures of synapse integrity both in vivo and in postmortem tissue, between AD + P and AD-P groups^15^. In the current report we provide a direct comparison of the PSD proteomes between AD + P and AD-P subjects, finding a shift to reduced levels of multiple proteins in the PSD of AD + P. Relative to AD-P, PSDs from AD + P subjects had lower levels of protein kinases which participate in a network that is also enriched for proteins with nominally significantly reduced levels in AD + P and for functions implicated in signaling to post-synaptic structural elements. The PSD protein reductions in AD + P relative to AD-P were not accounted for by the altered burden of neuropathologies or the greater reduction in excitatory neurons that we have previously reported in AD + P relative to AD-P. We were then able computationally identify several novel drug treatments predicted to reverse the PSD proteome signature of AD + P, and confirm that at least one of them, maraviroc, could reverse this signature in mouse cortex after short-term systemic administration.

Given the prominent role post-synaptic regulation of the actin cytoskeleton plays in the maintenance and plasticity of dendritic spines^18^, it is not surprising that the PSD proteome of AD + P was selectively depleted of proteins enriched for functions associated with actin regulation. The identification of impairments of this signaling network provides an opportunity for identifying compounds that might have specific therapeutic benefits for individuals with AD + P. We used a novel computational strategy, examining the overlap of gene knockout signatures with the AD + P PSD proteome alterations to identify upstream genes amenable to perturbation by existing compounds to potentially reverse the PSD proteome alterations in AD + P. We were able to confirm this prediction for maraviroc, which generated a net reversal of the PSD proteome reductions present in AD + P in a mouse model. Of note, our measure of net drug effect, the Signed Jaccard Index, showed a substantially larger effect in the PSD proteome (−0.01186) than that which was calculated using mRNA alterations induced by maraviroc in cultured neurons (−0.0009). The Signed Jaccard Index for the PSD proteome effect was similar to to the mean Signed Jaccard Index of −0.03264 that we reported for drugs indicated for treatment of CNS disease^20^.

While our computational prediction was confirmed for maraviroc, several caveats persist. Our validation was conducted in adult wildtype mice. Further tests in mouse models expressing AD-associated pathologies are warranted. Although there is no definitive mouse model for psychosis in AD^21^, the PSD proteome signature of at least some models of Aβ overproduction overlap with that of AD + P^16^. Even if maraviroc’s effects are further confirmed in AD models, a number of limitations may arise when repurposing a drug developed for treating other conditions. For example, any medication used will be applied to frail elderly individuals with multiple comorbidities, so preferred agents would be documented to be well-tolerated in such a population. Maraviroc, which was developed for chronic treatment of HIV infection, may well meet this tolerability benchmark, as might one of the other medications we identified, fostamatinib, which was developed for chronic use in populations with serious autoimmune disease^22,23^. Finally, we note that our confirmation for maraviroc need not hold true for the other identified compounds, which will require independent validation in intact neural model systems to evaluate their effects on the PSD.

Nevertheless, our strategy identified several genes that could represent novel targets for addressing the synaptic impairments in AD + P. For example, actin rod formation in neurons, which is promoted by agonism of the C-C Motif Chemokine Receptor 5 (CCR5*)*, the target of maraviroc, has been increasingly recognized as a component of Alzheimer’s neuropathology that induces PSD loss^24,25^. Likewise, treatment with maraviroc improved cognition in a small study of individuals with HIV-associated neurocognitive disorder^26^. Aurora Kinase B (AURKB*)*, the target by which fostamatinib was identified as a drug of potential benefit for AD + P, has recently been shown to play a role in promoting neurite formation during early neuronal development^27^ and to promote axonal repair after injury in post-mitotic neurons^28^.

Additionally, we identified reductions in levels of group of protein kinases that interact with a network that also includes other altered PSD proteins which serve to regulate the cytoskeleton. The roles of many of these kinases in the synaptic plasticity processes that regulate the maintenance of dendritic spines is well established (e.g. CAMK2A^29^, LIMK1^30^, typical^31^ and atypical^32^ PRKCs, TRIO^33^). Of interest, one of the atypical PRKCs, PRKCI, while not itself established as having a role in spine maintenance, was among the genes identified as nominally significantly associated with AD + P (Supplementary Data 6). We also identified TBK1, a kinase that when disrupted causes frontotemporal dementia/ALS and induces dendritic spine loss in animal models^34^. Albeit, whether TBK1 has a normative role in spine maintenance is not established. It is likely that not just PSD protein levels of these kinases, but their phosphorylation-dependent activation, and the extent of phosphorylation of their downstream targets, are altered in the PSD of AD + P relative to AD-P. Thus, an important future direction will be to delineate the phosphoproteome signature of the AD + P PSD. Such an investigation may both help to nominate candidate therapies directly targeting the altered kinases and may provide another metric to assess the therapeutic potential of other drug candidates.

We report the first study, to our knowledge, to directly interrogate the PSD in AD + P. The abundance of numerous PSD proteins was reduced in AD + P relative to both AD-P and comparison subjects. Examination of the PSD proteome signature of AD + P, relative to AD-P, revealed lower levels of an interacting set of protein kinases, regulators of Rho GTPases, and other protein regulators of the actin cytoskeleton. We were able to use the PSD protein signature of AD + P to computationally nominate several novel potential pharmacotherapies for the treatment of AD + P, and provide initial experimental validation that administration of one of the nominated drugs, maraviroc, to adult mice yielded a net reversal of the PSD protein signature of AD + P. Future studies are required to evaluate the ability of maraviroc and the other identified drugs to reverse the AD + P PSD proteome signature in animal models of AD-related pathologies and to delineate the phosphoproteome signature of the AD + P PSD.

## Methods

### Subjects

Tissue from subjects with AD were obtained from the brain bank of the Alzheimer Disease Research Center (ADRC) at the University of Pittsburgh, using protocols approved by the University of Pittsburgh Institutional Review Board and Committee for Oversight of Research and Clinical Training Involving Decedents. An initial group of 110 cases with a primary neuropathologic diagnosis of Alzheimer’s disease, a Braak stage between 3 and 5 were selected. End-stage cases, as defined by a Braak stage of 6, were excluded. Three AD cases were subsequently removed from analysis after QC of the proteomics data. A fourth case was discovered, prior to statistical analyses, to have not met inclusion criteria (did not meet neuropathologic criteria for AD) and was excluded, leaving a total of 106 AD subjects (Table 2).

**Table 2:** Subject Characteristics.

	AD – P (*n* = 47)	AD + P (*n* = 59)	Cognitively normal comparison (*n* = 19)	Overall *p* value
Age, years	84.4 (7.88)	83.7 (6.74)	84.5 (6.66)	0.826
Sex				
Male	19 (40.4%)	30 (50.8%)	6 (31.6%)	0.279
Female	28 (59.6%)	29 (49.2%)	13 (68.4%)	
PMI, hours	6.18 (3.73)	6.49 (4.22)	10.5 (6.63)	0.00163
Age of Onset, years	76.1 (8.33)	74.7 (7.03)		0.359
Duration of Illness, years	8.36 (3.63)	9.00 (3.25)		0.349
Braak Stage:				<0.001
0–II	0 (0%)	0 (0%)	19 (100%)	
III	9 (19.1%)	5 (8.5%)	0 (0%)	
IV	19 (40.4%)	22 (37.3%)	0 (0%)	
V	19 (40.4%)	32 (54.2%)	0 (0%)	
APOE-4				0.00383
Positive	25 (53.2%)	35 (59.3%)	3 (15.8%)	
Negative	22 (46.8%)	24 (40.7%)	16 (84.2%)	
Antipsychotic Use				
Yes	4 (8.5%)	11 (18.6%)	0 (0%)	0.0595
No	43 (91.5%)	48 (81.4%)	19 (100%)	

Results are reported as “mean (SD)” or as “n (% of group total)”. ANOVA was performed for continuous variables with post-hoc Tukey’s test. Chi-square (or as appropriate Fisher’s exact) tests were performed for all categorical variables, pairwise post-hoc tests used Benjamini-Hochberg correction to adjust for multiplicity. The overall p values for the 3-group comparisons are shown. For all variables that were significantly different, AD + P and AD-P groups were each significantly different from elderly cognitively normal comparison subjects. AD + P and AD-P did not significantly differ from each other on any variable. *For Age of Onset and Duration of Illness *p*-values were generated using a two-sample sample t-test.

Subjects underwent comprehensive evaluations by experienced clinicians in the ADRC, including neurologic, neuropsychological, and psychiatric assessments^16,17,35^. Using this information, information obtained from clinical records, and structured interviews with surviving relatives, an independent committee of experienced clinicians made consensus DSM-IV diagnoses for each subject. Psychosis was defined as the presence of delusions or hallucinations at any visit. Subjects with a preexisting psychotic disorder (e.g. schizophrenia) were excluded from the study.

Fixed and frozen tissue samples from 19 elderly cognitively normal comparison subjects from the Religious Order Study (ROS) were obtained from the Rush Alzheimer’s Disease Center (Table 2), along with basic demographic and neuropathologic information^36^. Mild neurodegenerative pathologic changes were accepted, up to a Braak stage of 2 for tau pathology, presence of sparse neuritic plaques or early TDP-43 pathology.

All subject samples were identified by a code number, and staff were blind to diagnosis group at all stages of sample preparation, data generation, and data extraction.

### Sample collection and neuropathologic assessment

For ADRC subjects, postmortem interval (PMI) was recorded at the time of brain removal. At autopsy, the brain was removed intact, examined grossly, and divided in the midsagittal plane. Gray matter samples from the right superior frontal gyrus of the dorsolateral prefrontal cortex (DLPFC) were dissected and frozen at −80 °C. The left hemibrain was immersion fixed in 10% buffered formalin for at least one week, sectioned into 1.0 cm coronal slabs, and sampled according to Consortium to Establish a Registry for Alzheimer’s Disease (CERAD) protocol for neuropathological diagnosis of AD^37^ or, since 2012, following National Institute of Aging-Alzheimer’s Association (NIA-AA) guidelines^38^. AD pathology was evaluated using the modified Bielschowsky silver stain^39^ and immunohistochemical staining for tau and amyloid β. Neuritic plaque density was assessed according to CERAD criteria^37^; distribution of tau pathology was classified according to Braak stages^40^. Lewy body pathology was assessed by alpha-synuclein immunohistochemistry, and positive cases were classified into amygdala-predominant, limbic/neocortical-predominant, or other categories, a modified scheme based on consensus criteria^38,41^. For analysis in this study, all alpha-synuclein positive categories were combined into one Lewy body positive group. Immunohistochemical staining for phospho-TDP-43 was performed on sections of amygdala, hippocampus, mesial temporal cortex and middle frontal gyrus^42^. Sections were evaluated for the absence or presence of TDP-43 positive neuronal cytoplasmic inclusions, neuronal intranuclear inclusions and dystrophic neurites. Based on the distribution of TDP-43 pathology, positive cases were classified into amygdala-predominant, mesial temporal, neocortical, or in cases when amygdala sections were not available, but all other sections were TDP-43 negative, indeterminate categories. For analysis in this study, all TDP-43 positive categories were combined into one TDP-43 positive group.

Assessment of vascular pathology included atherosclerosis of the circle of Willis, arteriolosclerosis in frontal white matter and cerebral amyloid angiopathy in DLPFC. Each was rated as none (0), mild (1), moderate (2), or severe (3), and a sum score was generated by adding the three individual scores. Microvascular lesions (MVL) were defined as remote microinfarcts/microhemorrhages not seen on gross examination and less than 1.0 cm in size. MVLs were enumerated in standardized sections^38^ of middle frontal gyrus (DLPFC), superior and middle temporal gyrus, inferior parietal lobule, occipital cortex (BA 17/18), basal ganglia at level of anterior commissure, and thalamus at the level of the subthalamic nucleus to create MVL counts.

Neuropathologic diagnoses of Alzheimer disease were made according to CERAD criteria^37^, although all AD subjects also met NIA-Reagan criteria^43^ for intermediate to high probability that their dementia was due to AD lesions.

For the 19 elderly cognitively normal comparison subjects from the ROS, the same variables were provided to us, including Braak stage, CERAD neuritic plaque scores, presence/absence of TDP-43 and Lewy body pathologies, severity of vascular pathologies, and diagnostic classification based on modified CERAD and NIA-Reagan criteria (applying criteria in the absence of a diagnosis of dementia)^44^.

### Biochemical fractionation and LC-MS/MS

Prior to biochemical fractionation, subjects were stratified into blocks of 10-11 subjects. Each block was balanced for diagnosis and sex. A post-hoc check ensured that the distributions of PMI, age, age of AD onset, Braak stage and APOE*ε4 carrier status did not differ between blocks. The order in which subject blocks were processed was randomized between successive stages of processing described below (i.e. between measurement of protein concentration, trypsin digestion, labeling with TMTPro, fractionation, and MS injection. PSD enrichments were generated using a variation on our previously described approach^45^. Grey matter was homogenized in Syn-PER reagent (Thermo Scientific, Waltham, MA); synaptosomes were prepared according to manufacturer’s protocol and washed with 1 ml 0.1 mM CaCl_2_. The washed pellet was resuspended in 500 µl of 20 mm Tris pH 8.0 with 1% Triton X-100, agitated on a rocker at 4 °C for 30 minutes, and centrifuged at 47,000 RPM for 30 min at 4 °C in the outer rim of a Sorval S80-AT2. The resulting pellet was washed with 250 µl 0.1 mM CaCl_2_ and centrifuged at 47,000 RPM for 30 min at 4 °C. The washed PSD pellet was taken up in 50 µl 1X S-Trap Buffer (100 mM TEAB, 5% SDS), vortexed, and bath sonicated. Total protein concentration was determined by Micro-BCA (Thermo Scientific).

A total of 10 µg total PSD protein from each sample were reduced, alkylated, and trypsin digested on S-Trap™ micro spin columns (ProtiFi) per manufacturer’s protocol. Subject blocks were randomly assigned to TMT blocks and labeled with TMTPro channels 1–11 as described in ref. ^46^. A pooled control was created using aliquots from the homogenate and synaptosome preparation steps which precede PSD generation (to save tissue resources). The pooled control was digested separately with S-Trap™ mini spin columns (ProtiFi, Farmingdale NY), split in two, and labeled with TMTPro channels 12 and 13. TMT labeled subject preparations from the same block were pooled along with 10 µg of the labeled pooled controls. The TMT labeled peptide pools were separated into eight fractions with the Pierce™ High pH Reversed-Phase Peptide Fractionation Kit (Thermo Scientific) per manufacture’s protocol, evaporated, and reconstituted in 20 µl 97% H2O, 3% ACN, 0.1% formic acid.

Approximately 1 µg of TMT labeled peptides was loaded onto a heated PepMap RSLC C18 2 µm, 100 angstrom, 75 µm × 50 cm column (ThermoScientific) and eluted over 180 min gradients optimized for each high pH reverse-phase fraction as in^47^. Sample eluate was electrosprayed (2000 V) into a Thermo Scientific Orbitrap Eclipse mass spectrometer for analysis. MS1 spectra were acquired at a resolving power of 120,000. MS2 spectra were acquired in the Ion Trap with CID (35%) in centroid mode. Real-time search (max search time = 34 s; max missed cleavages = 1; Xcorr = 1; dCn = 0.1; ppm = 5) was used for MS3. MS3 spectra were acquired in the Orbitrap with HCD (60%) with an isolation window = 0.7 m/z and a resolving power of 60,000, and a max injection time of 400 ms.

Raw MS files were processed in Proteome Discoverer version 2.5 (ThermoScientific). MS spectra were searched against the *Homo sapiens* SwissProt database. SEQUEST search engine was used (enzyme=trypsin, max. missed cleavage=2, min. peptide length=6, precursor tolerance=10ppm). Static modifications include acetylation (N-term, +42.011 Da), Met-loss (N-term, -131.040 Da), Met-loss+Acetyl (N-temr, −89.030 Da), and TMT labeling (N-term and K, +229.163 Da). Dynamic modification included oxidation (M, +15.995 Da). Peptide spectral matches were filtered by the Percolator node (max Delta Cn=0.05, target FDR (strict)=0.01, and target FDR (relaxed)=0.05). Reporter ion quantification was based on corrected S/N values with the following settings: integration tolerance=20ppm, method=most confident centroid, co-isolation threshold=100, and SPS mass matches=65.

### Quantitative immunohistochemistry

Serial 5 μm thick formalin-fixed, paraffin-embedded tissue sections were immunostained on an automated stainer (Discovery Ultra, Ventana, Tucson, AZ) using the following primary antibodies: PHF-1 (1:1000, kindly provided by Peter Davies), beta-amyloid NAB228 (1:4000 (Cell Signaling Technology, Danvers, MA), after 40 min pretreatment with 90% formic acid), and microglial markers Iba1 (1:500, Wako, Richmond, VA) and HLA-DR (1:100, Dako, Agilent Technologies, Santa Clara, CA). Except for beta-amyloid, slides for all other stains were pretreated with Discovery CC1 solution, a Tris-based buffer with a slightly basic pH (Ventana Medical Systems, Tucson, AZ). All slides were developed using a multimeric HRP/DAB detection system (Ventana Medical Systems, Tucson, AZ). No counterstaining was performed to ease signal quantification.

### Microscopy

Whole slide digital images of the immunostained sections were created using a Mirax MIDI slide scanner (Zeiss, Jena, Germany) or Aperio AT2 slide scanner (Leica, Deer Park, IL) at 40x resolution. A subset of cases was scanned on both scanners to confirm very high concordance of digital image analysis results. Digital image analysis was performed using NearCyte software (Andrew Lesniak, University of Pittsburgh). For each section, 4 rectangular regions of interest (ROI) of 4mm^2^ were created. These ROIs were defined to span the entire cortical thickness and were preferentially placed midway along the gyral axis to avoid tangentially cut cortical regions. Minor manual adjustments were made to adapt to curvatures and irregularities in the cortical ribbon. Once placed for the first analyzed stain (PHF-1), the same ROIs were re-used for all subsequent stains. If tissue folds or other artifacts prevented placement in the same location, the ROI was moved to an acceptable site as close as possible to the original location. For quantitative image analysis, thresholds for signal positivity were optimized manually for each stain and then maintained constant throughout the analysis of all slides. Signals from all four ROIs were integrated into two outcome variables: area ratio ( = positive area/entire field area) and mean signal intensity. For HLA-DR and Iba1 stains, an additional variable, the HLA-DR/Iba1 ratio was derived to normalize microglial activation (HLA-DR) to microglial density (Iba1). All analyses were done blinded to psychosis status.

### LC-MS/MS quality control and normalization

Zero abundance was treated as missing and missing rate was compared across samples. No obvious difference was found across plexes or diagnosis groups (Fig. S6). Then we performed rigorous QC and normalization on all quantified peptides. First, we performed sample loading normalization to make the total abundance the same across all samples. Second, peptides that were missing in both pooled control samples in at least half of the plexes were removed. We also removed peptides that were mapped to multiple proteins or genes. Three AD subjects who were included in the assay were deemed as outliers based on their total abundances (Fig. S7a, b) and they were excluded from the subsequent data normalization steps and analyses. Next, an internal reference scaling (IRS) normalization^48^ was performed for each peptide, where the scaling factor (for each plex) was calculated as the ratio of the overall mean of all pooled samples to the mean of the within-plex pooled samples. Finally, a median normalization was performed to make the median of each sample equal to the overall median of all samples (Fig. S7c). Post QC we quantified 27,919 peptides with a missing rate ≤50% across all samples and 5,807 peptides for which all peptides were quantified in all samples (i.e. missing rate = 0%).

One additional subject who was included in the above normalizations was discovered, prior to statistical analysis, to have not met inclusion criteria (did not meet neuropath criteria for AD) and was excluded from all subsequent analyses, leaving a final cohort for analysis of 106 AD subjects and a reference group of 19 normal comparison subjects (Table 2).

### Statistical analysis

Sample size was determined by power analysis of data from our prior publication^16^. Power was a function of sample size and pi, where pi is the proportion of altered protein ratios between AD-P and AD + P. Assumptions for the power analysis were that the average ratio for the pi proteins would be = 1.17 (and for the 1-pi proteins would = 1.0), SD (on log2 protein level) = 1.0, and one-sided alpha = 0.05. Based on these criteria, we projected a sample size between 90 and 120 AD subjects would yield power of 0.75–0.84.

To test PSD yield between groups we performed linear regression. To test protein abundance, for each sample we multiplied the PSD yield to the normalized peptide abundance to account for the PSD yield variation in samples. Next, we performed PeCorA^49^ analysis to identify “uncorrelated” peptides within each protein. In the end, we rolled up yield-adjusted peptide data to protein level by averaging the z-score of each peptide (on the log2 scale) that is mapped to the same protein. The “uncorrelated” peptides were treated as separate proteins and were not rolled up. Using a present call threshold of 100% when selecting peptides in the roll-up step resulted in 1613 proteins for analysis, and a threshold of 50% yielded 4025 proteins. These values compare favorably with recent studies reporting 6,533–8,619 proteins with a missing rate ≤50% assayed using similar methods (albeit with a deeper off-line fractionation) in the more complex total gray matter proteome in AD^19,50^. Then linear regression was performed using the Limma package^51,52^ in R (version 4.1.0) for each yield-adjusted protein.

For tests of our primary comparison between AD + P and AD-P, all analyses adjusted for age, PMI, sex, APOE*ε4 carrier status, Lewy body positivity, and phospho-tau area ratio (log2 transformed). For comparisons of the AD groups to our reference group of cognitively normal elderly subjects, analyses were adjusted for age, PMI, and sex. APOE*ε4 carrier status and neuropathology variables were excluded from these comparisons as they are highly associated with diagnosis comparing AD and control. In all analyses two-group model-based log2 of fold change between any two groups was calculated for each protein. All statistical tests performed are two-sided. The Benjamini-Hochberg method was used to yield FDR-adjusted p-values.

Functional annotation clustering analysis of differentially expressed proteins was performed with DAVID^53^, using the default settings. In each analysis the nominally significantly differentially expressed proteins were tested for enrichment relative to a background of the 1613 proteins quantified in all samples (or in supplemental analyses a background of the 4025 proteins with missing rate <50%).

### Computational systems pharmacology

Since all of the nominally significant differentially expressed proteins were reduced in AD + P relative to AD-P, medications that directly target these proteins may have reduced efficacy due to the lower levels of the target. Therefore, we utilized a strategy designed to identify upstream targets that regulate the expression of these nominally differentially expressed proteins.

The Illumina correlation engine (https://hsls.ce.basespace.illumina.com/c/nextbio.nb) knockdown atlas was used to identify genes which, when knocked down, altered expression of the mRNA corresponding to the 1613 proteins quantified in 100% of AD subjects. The identified genes (upstream-genes) were then extracted, as were the correlations of their knockdown transcriptome signature with the directions of alteration of the 1613 proteins.

To identify medications that target the upstream-genes, information about medications and their targets were extracted from DrugBank (https://www.drugbank.ca/)^54^ including medication names, targets of medications and their corresponding actions. The desired drug-target action was identified by aligning the correlation between upstream-gene knockdown and our dataset. For example, if an upstream-gene was negatively correlated with our dataset, it means that its knockout recapitulated many of the alterations we observed in AD + P relative to AD-P. Therefore, drugs that antagonize or otherwise inhibit its activity would be predicted to induce a signal that can reverse the expression profile we observed in AD + P, which may lead to beneficial effects.

To confirm whether our identified drug candidates themselves would result in reversing the AD + P PSD proteome signature, we extracted the gene expression profile for each drug from Level 5 LINCS L1000 data^55^, a collection of gene expression profiles for thousands of perturbagens at a variety of time points, doses, and cell lines (GEO database accession numbers: GSE70138 and GSE92742). The gene expression profiles were included only if they were from drug treatments on a cell line derived from the central nervous system and the drug dose was ≥ 1 µM. To identify genes that were significantly differentially expressed, the Z scores from multiple tests for a same gene were averaged. An average |Z | > 1 was considered a significant effect^56^.

The association between drug and PSD data was quantitatively evaluated with the Signed Jaccard Index^20^. The index ranges from +1 to −1, where +1 and −1 indicate the same, or inverse, pattern of two gene sets.

### Maraviroc effects on the PSD proteome

Maraviroc was dissolved in a vehicle solution of 18% ethanol in normal saline. Twelve 3mo old C57Bl/6 J WT mice (6 males and 6 females) were randomized to daily intraperitoneal injection of maraviroc 50 mg/kg or an equivalent volume of vehicle, balanced for sex and with the experimenter blind to injectate identity. Mice were sacrificed 2 hours post the last daily injection, to approximate Tmax, and the right cerebral cortex was harvested on dry ice for proteomics. Biochemical Fractionation and LC-MS/MS were as described above. Sample QC was as described above. One maraviroc-treated mouse was identified as having protein yield (0.35 µg/µl) much lower than the mean yield of the remaining mice (1.31 µg/µl). It was confirmed to be an outlier when examining the resultant peptide abundances as part of the sample QC (Fig. S8) and was excluded from analyses, leaving a final N of 5 maraviroc treated and 6 vehicle treated mice. Post QC a total of 22,965 peptides with 100% present calls were rolled-up to generate measures of 3,852 proteins (Supplementary Data 10), 1370 of which overlapped with the 1613 proteins measured in the PSD of AD subjects.

Experiments were conducted in adherence to the National Institutes of Health guidelines for laboratory animal care and were approved by the Institutional Animal Care and Use Committee at the University of Pittsburgh.

### Reporting summary

Further information on research design is available in the Nature Portfolio Reporting Summary linked to this article.

## Supplementary information

Supplemental Material

Description of Additional Supplementary Files

Supplementary Data 1

Supplementary Data 2

Supplementary Data 3

Supplementary Data 4

Supplementary Data 5

Supplementary Data 6

Supplementary Data 7

Supplementary Data 8

Supplementary Data 9

Supplementary Data 10

Reporting Summary

## Data Availability

The mass spectrometry proteomics data have been deposited to the ProteomeXchange Consortium via the PRIDE^57^ partner repository with the dataset identifiers PXD042025 10.6019/PXD042025 and PXD042026 10.6019/PXD042026. All other data are available from the corresponding author (or other sources, as applicable) on reasonable request.
