# Supplementary material for: Targeting the post-synaptic proteome has therapeutic potential for psychosis in Alzheimer Disease
Source: Commun Biol. 2023 Jun 2;6:598. doi: 10.1038/s42003-023-04961-5 (PMC10238472; doi:10.1038/s42003-023-04961-5)
Supplement: Supplementary file 1 — Supplemental Material [file 42003_2023_4961_MOESM1_ESM.pdf]

SUPPLEMENTAL MATERIALS

S1. Supplemental Discussion of *Overlap with GWAS findings*

We additionally identified an enrichment of genes encoding actin binding proteins, *SYNPO*, *SYNE1*, *ALDOA*, and *VPS16*, when we examined the overlap of the AD+P PSD proteome signature with genes nominated by our GWAS of AD+P. Two of these, *SYNPO* and *SYNE1*, are implicated in processes serving dendritic spine plasticity and maintenance, providing a link between the genetics of AD+P and its biologic signature of post-synaptic deficits. *SYNPO* encodes the protein synaptopodin, a dendritic spine protein that contributes to maintaining the long-term stability of large dendritic spines [1]. *SYNE1* encodes multiple actin-binding proteins via extensive alternative splicing. One of these proteins, candidate plasticity gene 2 (CPG2), which is transcribed from the region of *SYNE1* associated with risk for bipolar disorder [2], serves as a bridge between the dendritic spine endocytic machinery and the actin cytoskeleton necessary for activity dependent endocytosis of glutamate receptors [3]. *ALDOA* encodes the glycolytic enzyme Aldolase, Fructose-Bisphosphate A protein. While shown to promote lung cancer metastasis by interacting with gamma-actin [4], its role in neuronal function remans obscure. Of interest, however, given our computational predictions, ALDOA protein is found in actin rods [5]. *VPS16* encodes the VPS16 Core Subunit of CORVET and HOPS Complexes protein, which contributes to autophagosome-lysosome fusion. Mutations in *VPS16* cause dystonia syndromes, though the functions of *VPS16* in neurons, and the functional consequences of the identified mutations for neuronal and postsynaptic function are not currently established [6]. *TBC1D10B* encodes a GTPase-activating protein for members of the Rab family,[7] where it participates in an endosomal-lysosome pathway that is important in the clearance of tau protein.[8]

In our recent genome-wide association meta-analysis of over 12,000 AD subjects with or without psychosis, the most significant genetic locus associated with psychosis in AD was contained in a gene encoding ectonucleotide pyrophosphatase/phosphodiesterase 6 (ENPP6) [9]. One other locus, in *SUMF1*, also reached genome-wide significance. We observed a reduced level of ENPP6 in AD+P relative to AD-P [Log_2(AD+P/AD-P) = -0.40] that was just outside the 240 PSD proteins with nominally reduced abundance (P=0.051, **Supplementary Data 1**). Formylglycine generating enzyme, the protein product of *SUMF1*, was not detected in our assay, The expression of ENPP6 is selective for the oligodendrocyte lineage [10], peaking as oligodendrocyte precursor cells (OPCs) differentiate into mature oligodendrocytes [11]. It has been established for some time that presynaptic terminals of excitatory neurons containing synaptic vesicles and release machinery align at electron-dense synaptic clefts abutting the processes of OPCs, and that the latter closely resemble the PSDs of dendritic spines, containing a postsynaptic membrane specialization and glutamate receptors (reviewed in[12]). Though not established, it is likely that these OPC PSDs are co-isolated with neuronal PSDs in our enrichment procedure. Others have demonstrated that synaptic input from glutamatergic neurons onto AMPA and NMDA receptors within the "PSDs" of oligodendrocytes regulate OPC proliferation and differentiation of OPCs to mature oligodendrocytes [13,14], a process during which ENPP6 expression is at its peak, and which is required for learning complex tasks [11]. Our finding of reduced ENPP6 abundance in the PSDs of AD+P would therefore suggest that activity dependent maturation of OPCs is impaired in AD+P relative to AD-P, and may contribute to the greater cognitive impairment in AD+P. Whether, and by what mechanism, this may relate to the genetic association of *ENPP6* with AD+P risk remains an open question.

S2. Supplemental Figures

Figure S1. PSD Enrichment in AD. We validated our approach to PSD enrichment in DLPFC of 4 AD cases drawn from the current cohort. PSD preparation was as described in ***Biochemical Fractionation and LC-MS/MS***, with the following minor differences. 8 μg total PSD and corresponding homogenate protein were digested, TMT labeled in a single block, and separated into 9 fractions prior to LC-MS injection. Enrichments (and depletions) relative to homogenate of canonical PSD (GRIA2, GRIN1, DLG4), presynaptic (VAMP2, SNAP25), and cytosolic (GLUL, DPYSL2) constituents are shown. Dashed line indicates no change (Log2 scale) relative to the homogenate. Error bars represent SD.

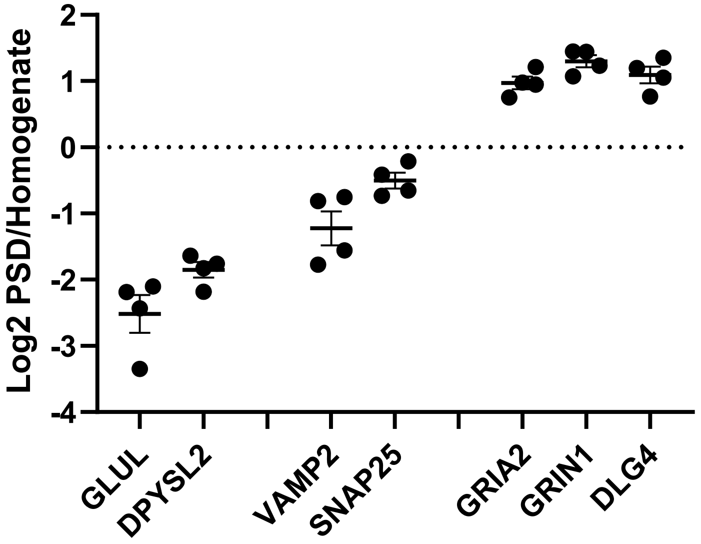

Figure S2. PSD Yields of AD+P, AD-P, and cognitively normal elderly reference subjects. Yields are shown as μg of PSD protein per μg of gray matter protein. Mean yield of AD+P group is lower than mean yield of AD-P group ($t = -2.17$, $df = 98$, $p = 0.033$) and lower than elderly cognitively normal reference subjects ($t = -2.36$, $df = 119$, $p = 0.020$). Mean yield of AD-P subjects is not significantly lower than that of the elderly cognitively normal reference subjects ($t = -0.78$, $df = 119$, $p = 0.433$).

Group N=125	Min	Mean	SD	Max
Control N=19	0.240	0.526	0.201	0.896
AD-P N=47	0.120	0.513	0.241	1.294
AD+P N=59	0.135	0.421	0.192	1.058

Figure S3. Follow up tests of distribution of levels of PSD proteins quantified in DLPFC of AD+P and AD-P subjects. **(a)** Distribution of levels of 4,025 PSD proteins quantified in DLPFC of AD+P and AD-P subjects using a peptide present call threshold of 50%. (**b-c)** We previously reported that excitatory neuron proportion is lower in DLPFC of AD+P relative to AD-P in a cohort of subjects, 76 of whom overlap with subjects in the current study [15]. The distribution of levels of 1,613 PSD proteins quantified in DLPFC of the 76 AD+P and AD-P overlapping subjects without (**b**) and with (**c**) covarying for excitatory neuron proportion. Distributions of $\log_2$ ratios are shown for all proteins, adjusted for covariates, Age, PMI, Sex, *APOE**E4, Lewy Body presence, and phospho-Tau area fraction. The dashed vertical lines represent no difference in the ratio of protein levels between groups. In all panels AD+P is characterized by a significant shift towards lower PSD protein levels compared to AD-P. Black points indicate proteins with nominally significant differences in levels in AD+P relative to AD-P ($p<0.05$).

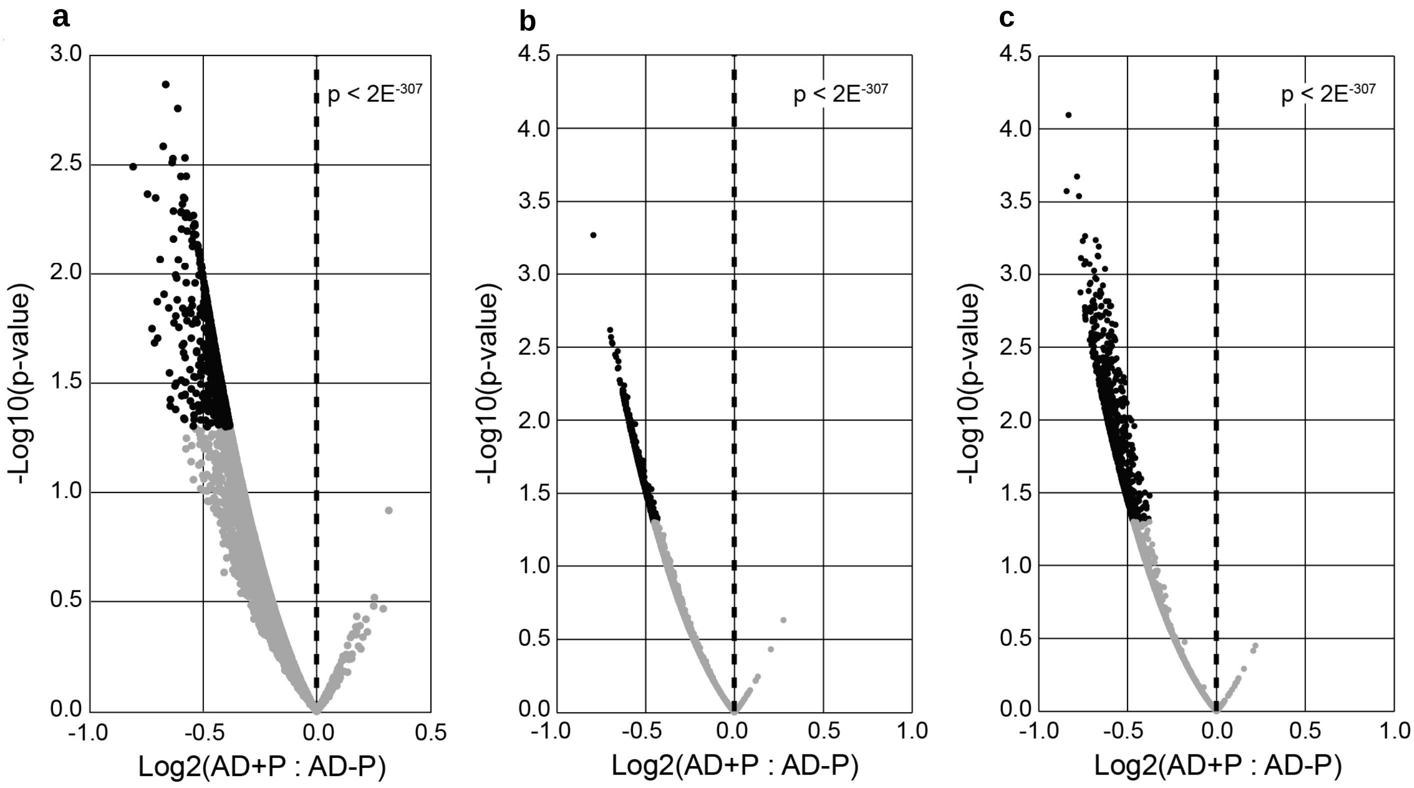

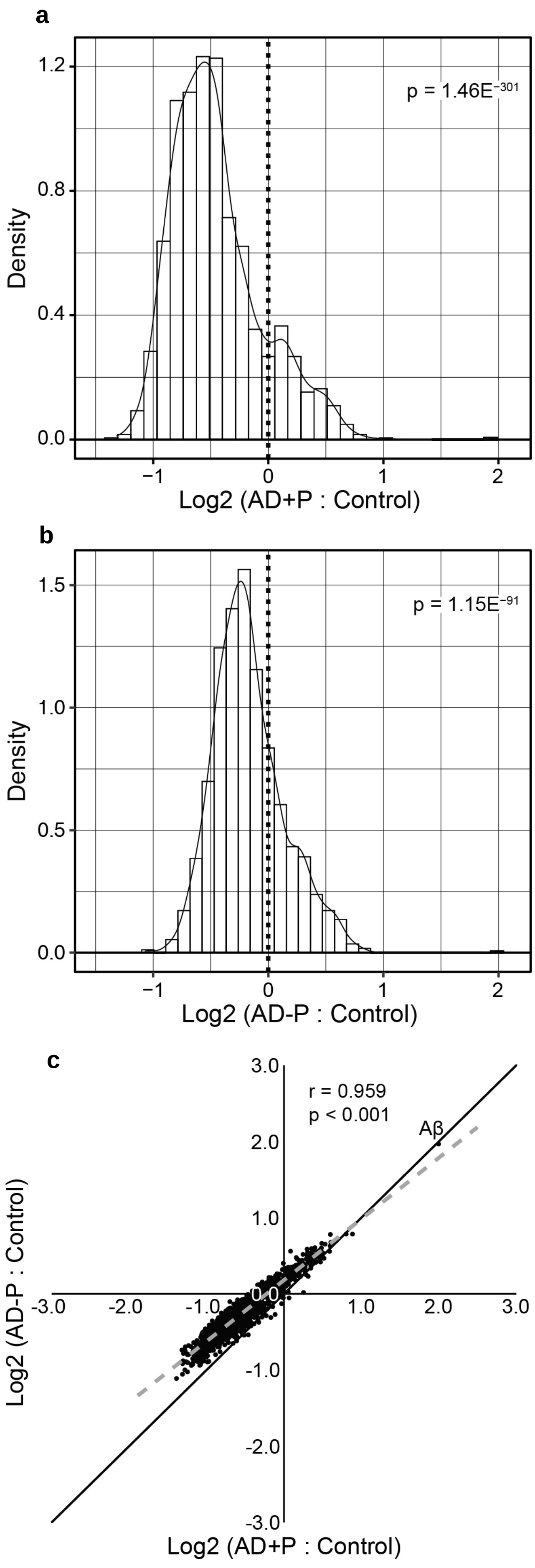

Figure S4. Distribution of levels of 1,613 PSD proteins quantified in DLPFC using peptides present in 100% of AD+P and AD-P subjects relative to cognitively normal elderly comparison subjects. **(a&b)** Distributions of Log2 ratios are shown for all proteins, adjusted for covariates, Age, PMI, and Sex. The dashed vertical line represents no difference in the ratio of protein levels between groups. Both AD groups are characterized by a significant shift towards lower PSD protein levels compared to cognitively normal elderly controls, with greater reductions in AD+P (**a**) than in AD-P (**b**) (as also seen in **Figure 2**). **(c)** The PSD alterations in the two AD groups relative to control are highly correlated (dashed line, $r=0.95$, $p<0.001$). Nevertheless, the greater reductions in AD+P than AD-P relative to control are evident as points above the solid unity line ($y=x$). The protein most elevated in both AD+P and AD-P relative to comparison subjects was APP, due solely to the increased levels of two peptides (RHDSGYEVHHQK and KLVFFAEDVGSNK) found within the Aβ sequence (noted as Aβ).

Figure S5. Comparison of functional enrichment of differentially expressed PSD proteins in AD+P and AD-P relative to cognitively normal elderly comparison subjects with prior report of functional enrichment of AD gray matter homogenate relative to control. Protein co-expression modules for AD vs Control are from [16] in which modules were functionally characterized if they demonstrated a nominally significant ($p<0.05$) correlation with NIA Reagan Score. Redundant or ambiguous functional characterizations of the modules are not shown. From the current report, all AD+P and AD-P PSD functional enrichment clusters relative to controls with enrichment score >=1 were included (see **Tables S5A** and **S5B**). N indicates total number of functions annotated, numbers in color indicate overlapping functions and match text colors in the table.

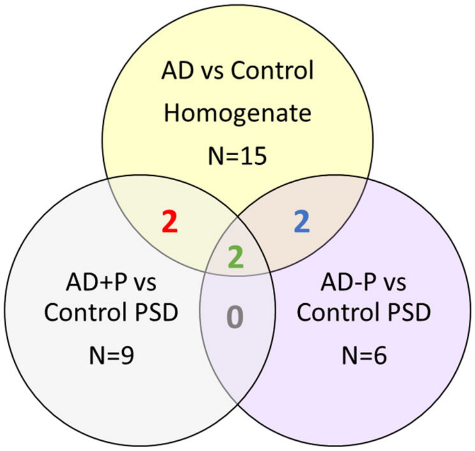

AD vs Control	AD+P vs Control	AD-P vs Control
Ribosome	GO:0003735~structural constituent of ribosome	GO:0005840~ribosome
Glycosylation/ER	KW-0325~Glycoprotein	KW-0325~Glycoprotein
Synapse/Neuron	KW-0770~Synapse	KW-1133~Transmembrane helix
Mitochondria	IPR023395:Mitochondrial carrier domain	IPR001806:Small GTPase superfamily
Cell-ECM Interaction	KW-0221~Differentiation	GO:0007155~cell adhesion
Ubiquitination	IPR000008:C2 calcium-dependent membrane targeting	GO:1990948~ubiquitin ligase inhibitor activity
Sugar Metabolism	GO:0000226~microtubule cytoskeleton organization	
RNA Splicing	KW-0375~Hydrogen ion transport	
Golgi	GO:0005516~calmodulin binding	
Protein Transport		
Axonogenesis		
MAPK/Metabolism		
MHC Complex/Immune		
Oligo/Myelination		
Post-Synaptic Density		

Figure S6. Per-sample peptide missing rate (before QC or normalization). Note that zero abundance was treated as missing. No obvious difference in missing rate is observed across plexes/batches or diagnosis groups. The missing rate for each sample is close to the average missing rate (0.578).

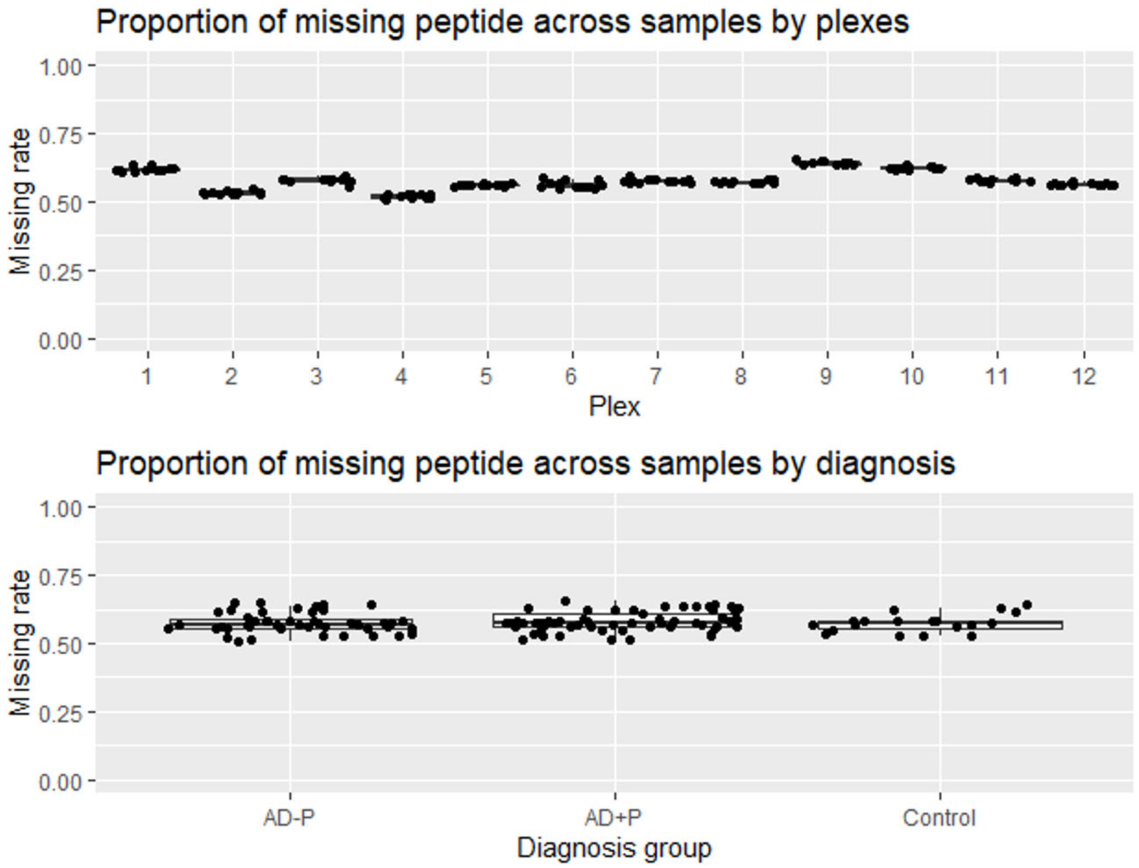

Figure S7. Outlier identification and peptide normalization. (a) Identification of subject outliers excluded prior to peptide intensity normalization and statistical analysis. Left shows box plot of total peptide abundance for each TMT plex. Three extreme outlying subjects are denoted by the blue arrows, which indicate their corresponding locations in the multidimensional scaling plot, where they are also seen to separate from the main cluster of subjects (Right). **(b)** Box plot of total peptide abundance for each TMT plex and the multidimensional scaling plot (in log scale) after removing the outliers. There is a potential plex effect, for example, purple plex (plex 10) located in the bottom right corner (Right) **(c)** Box plot of total peptide abundance for each TMT plex and the multidimensional scaling plot (in log scale) after normalization. After normalization, total peptide abundances for each TMT plex are similar (Left) and there is no obvious plex effect (Right).

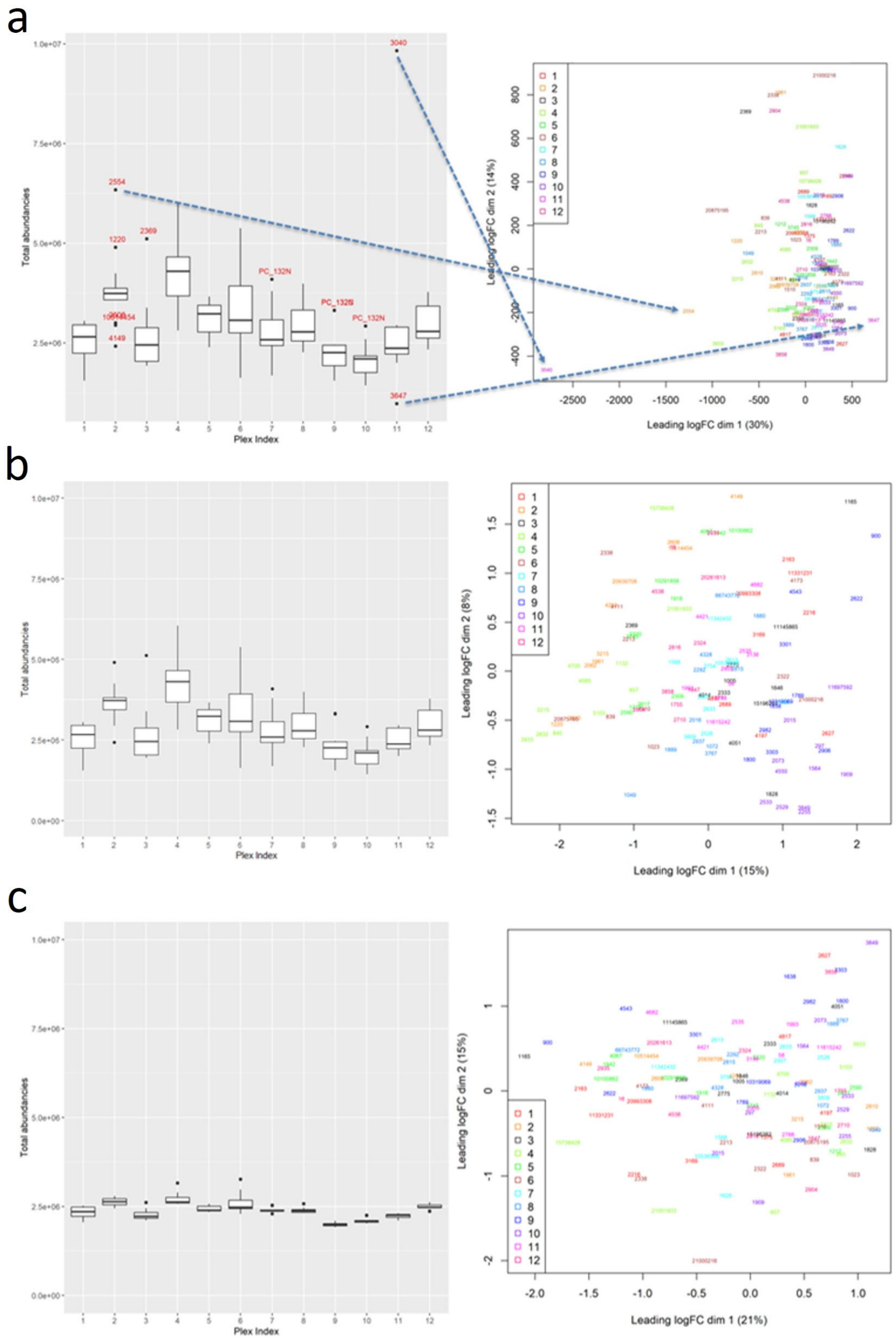

Figure S8. Outlier identification in maraviroc-treated mice. (a) Arrow identifies maraviroc-treated mouse with low PSD protein yield (solid dot). **(b)** Multidimensional scaling plot using as input all proteins quantified with peptides having 100% present calls confirms the same mouse (arrow) as an outlier which was removed from subsequent analyses.

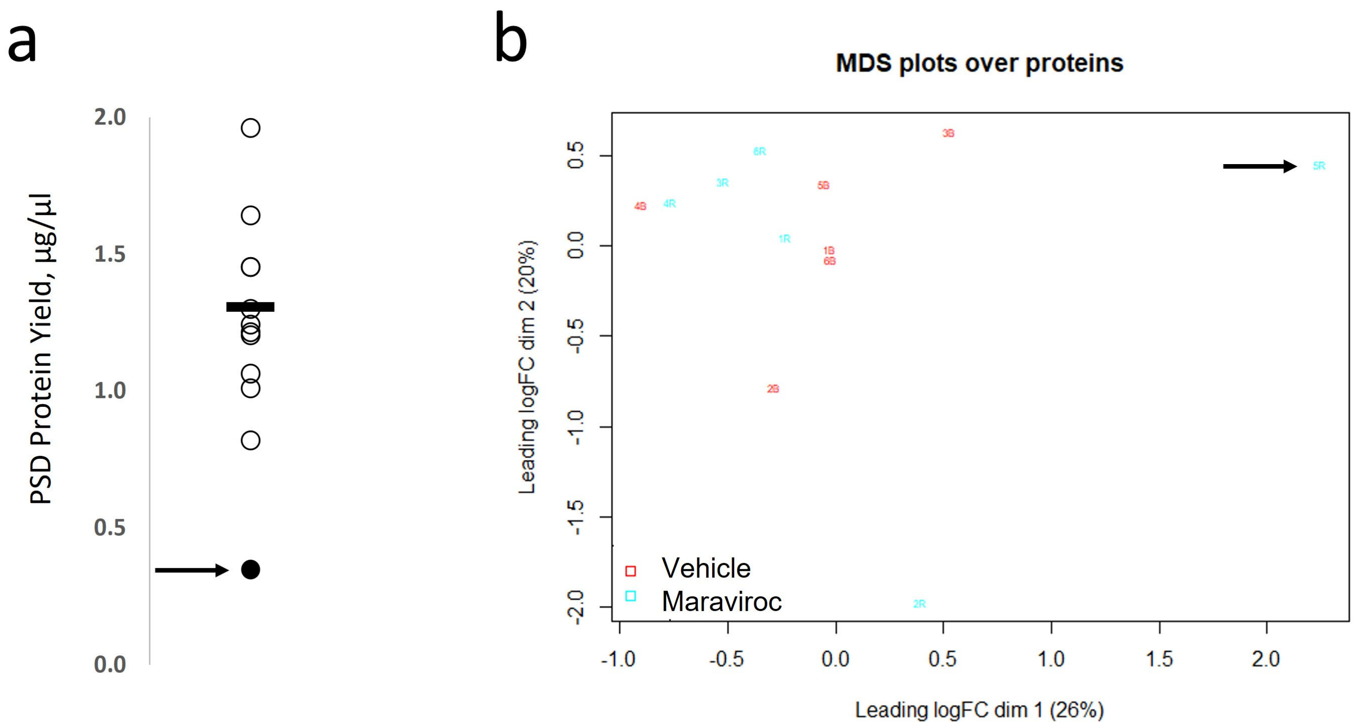

S4. Supplemental References

1. Yap, K., *et al.* The actin-modulating protein synaptopodin mediates long-term survival of dendritic spines. *Elife* **9**(2020).
2. Rathje, M., *et al.* Genetic variants in the bipolar disorder risk locus SYNE1 that affect CPG2 expression and protein function. *Mol Psychiatry* **26**, 508-523 (2021).
3. Loebrich, S., *et al.* CPG2 Recruits Endophilin B2 to the Cytoskeleton for Activity-Dependent Endocytosis of Synaptic Glutamate Receptors. *Curr Biol* **26**, 296-308 (2016).
4. Chang, Y.C., *et al.* Therapeutic Targeting of Aldolase A Interactions Inhibits Lung Cancer Metastasis and Prolongs Survival. *Cancer Res* **79**, 4754-4766 (2019).
5. Walter, L.M., Rademacher, S., Pich, A. & Claus, P. Profilin2 regulates actin rod assembly in neuronal cells. *Sci Rep* **11**, 10287 (2021).
6. Steel, D., *et al.* Loss-of-Function Variants in HOPS Complex Genes VPS16 and VPS41 Cause Early Onset Dystonia Associated with Lysosomal Abnormalities. *Ann Neurol* **88**, 867-877 (2020).
7. Ishibashi, K., Kanno, E., Itoh, T. & Fukuda, M. Identification and characterization of a novel Tre-2/Bub2/Cdc16 (TBC) protein that possesses Rab3A-GAP activity. *Genes Cells* **14**, 41-52 (2009).
8. Lin, H., Deaton, C.A. & Johnson, G.V.W. Commentary: BAG3 as a Mediator of Endosome Function and Tau Clearance. *Neuroscience* (2022).
9. DeMichele-Sweet, M.A.A., *et al.* Genome-wide association identifies the first risk loci for psychosis in Alzheimer disease. *Mol Psychiatry* **26**, 5797-5811 (2021).
10. Zhang, Y., *et al.* An RNA-sequencing transcriptome and splicing database of glia, neurons, and vascular cells of the cerebral cortex. *J Neurosci* **34**, 11929-11947 (2014).
11. Xiao, L., *et al.* Rapid production of new oligodendrocytes is required in the earliest stages of motor-skill learning. *Nat Neurosci* **19**, 1210-1217 (2016).
12. Kula, B., Chen, T.J. & Kukley, M. Glutamatergic signaling between neurons and oligodendrocyte lineage cells: Is it synaptic or non-synaptic? *Glia* **67**, 2071-2091 (2019).
13. Gallo, V., *et al.* Oligodendrocyte progenitor cell proliferation and lineage progression are regulated by glutamate receptor-mediated K+ channel block. *J Neurosci* **16**, 2659-2670 (1996).
14. Li, C., *et al.* A functional role of NMDA receptor in regulating the differentiation of oligodendrocyte precursor cells and remyelination. *Glia* **61**, 732-749 (2013).
15. DeChellis-Marks, M.R., *et al.* Psychosis in Alzheimer's Disease Is Associated With Increased Excitatory Neuron Vulnerability and Post-transcriptional Mechanisms Altering Synaptic Protein Levels. *Frontiers in Neurology* **13**(2022).
16. Johnson, E.C.B., *et al.* Large-scale deep multi-layer analysis of Alzheimer's disease brain reveals strong proteomic disease-related changes not observed at the RNA level. *Nat Neurosci* **25**, 213-225 (2022).
