# Supplementary material for: Targeting the post-synaptic proteome has therapeutic potential for psychosis in Alzheimer Disease
Source: Commun Biol. 2023 Jun 2;6:598. doi: 10.1038/s42003-023-04961-5 (PMC10238472; doi:10.1038/s42003-023-04961-5)
Supplement: Supplementary file 2 — Description of Additional Supplementary Files [file 42003_2023_4961_MOESM2_ESM.pdf]

Description of Additional Supplementary Files

File Name: Supplementary Data 1

Description: Comparison of AD subjects with and without psychosis for 1613 proteins quantified using peptides present in 100% of cases.

File Name: Supplementary Data 2

Description: Functional annotation of proteins with nominally significant (p<0.05) differences between AD+P and AD-P (N=240 proteins) relative to a background of the 1613 proteins measured in all subjects. Only results for the clusters with enrichment scores >=1.0 are shown.

File Name: Supplementary Data 3

Description: Comparison of AD subjects with and without psychosis for 4026 proteins quantified using peptides present in $\geq$ 50% of cases. S3A- Differential Expression. S3B- Functional Annotation Analysis

File Name: Supplementary Data 4

Comparison of AD subjects with and without psychosis to cognitively normal elderly reference subjects for 1613 proteins quantified using peptides present in 100% of cases.

File Name: Supplementary Data 5

Description: Functional annotation of proteins, excluding APP, with nominally significant (p<0.05) differences versus cognitively normal elderly reference subjects for AD+P (N=976 proteins) and AD-P (N=324 proteins) relative to a background of the 1612 proteins (excluding APP) measured in all subjects. Only results for the clusters with enrichment scores >=1.0 are shown.

File Name: Supplementary Data 6

Description: Comparison of proteins present in 100% of cases with nominally significant (p<0.05) associations in the current comparison of AD with and without psychosis with previously reported gene-based tests of association. Results are shown for 239 of the 240 proteins, as P12532 mapped to more than one gene.

File Name: Supplementary Data 7

Description: Potential regulator genes identified by correlation of their gene knockdown transcriptome signature with the PSD protein signature of AD+P.

File Name: Supplementary Data 8

Description: Drug gene signatures. S8A. Data sets queried. S8B. Gene signature results.

File Name: Supplementary Data 9

Description: Effects of Maraviroc on the 1370 PSD proteins also measured in AD+P.

File Name: Supplementary Data 10

Description: Comparison of Maraviroc and vehicle treated mice for 3851 proteins quantified using peptides present in 100% of cases.
