# Supplementary material for: Targeting the post-synaptic proteome has therapeutic potential for psychosis in Alzheimer Disease
Source: Commun Biol. 2023 Jun 2;6:598. doi: 10.1038/s42003-023-04961-5 (PMC10238472; doi:10.1038/s42003-023-04961-5)
Supplement: Supplementary file 13 — Reporting Summary [file 42003_2023_4961_MOESM13_ESM.pdf]

nature portfolio

Corresponding author(s): Robert Sweet

Last updated by author(s): Apr 28, 2023

Reporting Summary

Nature Portfolio wishes to improve the reproducibility of the work that we publish. This form provides structure for consistency and transparency in reporting. For further information on Nature Portfolio policies, see our Editorial Policies and the Editorial Policy Checklist.

Statistics

For all statistical analyses, confirm that the following items are present in the figure legend, table legend, main text, or Methods section.

n/a	Confirmed	
☐	☒	The exact sample size (n) for each experimental group/condition, given as a discrete number and unit of measurement
☐	☒	A statement on whether measurements were taken from distinct samples or whether the same sample was measured repeatedly
☐	☒	The statistical test(s) used AND whether they are one- or two-sided<br>*Only common tests should be described solely by name; describe more complex techniques in the Methods section.*
☐	☒	A description of all covariates tested
☐	☒	A description of any assumptions or corrections, such as tests of normality and adjustment for multiple comparisons
☒	☐	A full description of the statistical parameters including central tendency (e.g. means) or other basic estimates (e.g. regression coefficient) AND variation (e.g. standard deviation) or associated estimates of uncertainty (e.g. confidence intervals)
☐	☒	For null hypothesis testing, the test statistic (e.g. F, t, r) with confidence intervals, effect sizes, degrees of freedom and P value noted<br>*Give P values as exact values whenever suitable.*
☒	☐	For Bayesian analysis, information on the choice of priors and Markov chain Monte Carlo settings
☒	☐	For hierarchical and complex designs, identification of the appropriate level for tests and full reporting of outcomes
☐	☒	Estimates of effect sizes (e.g. Cohen's d, Pearson's r), indicating how they were calculated

Our web collection on statistics for biologists contains articles on many of the points above.

Software and code

Policy information about availability of computer code

Data collection	MS Data was collected using Proteome Discoverer V2.5
Data analysis	R (version 4.1.0) was used to analyze the data. The main analyses were done using the Limma package.

For manuscripts utilizing custom algorithms or software that are central to the research but not yet described in published literature, software must be made available to editors and reviewers. We strongly encourage code deposition in a community repository (e.g. GitHub). See the Nature Portfolio guidelines for submitting code & software for further information.

Data

Policy information about availability of data

All manuscripts must include a data availability statement. This statement should provide the following information, where applicable:

- Accession codes, unique identifiers, or web links for publicly available datasets
- A description of any restrictions on data availability
- For clinical datasets or third party data, please ensure that the statement adheres to our policy

Proteomic data will be made available to the scientific community at the latest at the time of publication via deposition in the Pride Database

Field-specific reporting

Life sciences study design

All studies must disclose on these points even when the disclosure is negative.

Sample size	Sample size was determined by power analysis of data from our prior publication (Krivinko et al. 2018). Power was a function of sample size and pi, where pi is the proportion of altered protein ratios between AD-P and AD+P. Assumptions for the power analysis were that the average ratio for the pi proteins would be = 1.17 (and for the 1-pi proteins would = 1.0), SD (on log2 protein level) = 1.0 , and one-sided alpha=0.05. Based on these criteria, we projected a sample size between 90-120 AD subjects would yield power of 0.75-0.84
Data exclusions	One subject who was included in sample preparation and LC-MS assay was discovered, prior to statistical analysis, to have not met inclusion criteria (did not meet neuropath criteria for AD). Because this sample was in the LC-MS assay, it was used in the data normalization, but excluded from all subsequent analyses.
Replication	We conducted a split sample analysis, confirming our primary finding of down-regulation of PSD protein levels in AD+P persisted when examining both the subset of 51 individuals who were included in our prior report of reduced levels of a targeted panel of 190 synaptic proteins (Krivinko et al. 2018), or the remaining 55 AD subjects who were newly evaluated (both p<1.5E-286, not shown in manuscript).
Randomization	Prior to biochemical fractionation, subjects were stratified into blocks of 10-11 subjects, each block was balanced for diagnosis and sex. A post-hoc check also ensured that the distributions of PMI, age, age of AD onset, Braak stage and APOE*ε4 carrier status did not differ among all 12 blocks. The order in which subject blocks were processed was randomized between successive stages of processing, i.e. between measurement of protein concentration, trypsin digestion, labeling with TMTPro channels 1-11, fractionation, and MS injection.
Blinding	All subject samples were identified by a code number, and staff were blind to diagnosis group, at all stages of sample preparation, data generation, and data extraction.

Reporting for specific materials, systems and methods

We require information from authors about some types of materials, experimental systems and methods used in many studies. Here, indicate whether each material, system or method listed is relevant to your study. If you are not sure if a list item applies to your research, read the appropriate section before selecting a response.

Materials & experimental systems

n/a	Involved in the study	
☒	☐	Antibodies
☒	☐	Eukaryotic cell lines
☒	☐	Palaeontology and archaeology
☐	☒	Animals and other organisms
☐	☒	Human research participants
☐	☒	Clinical data
☒	☐	Dual use research of concern

Methods

n/a	Involved in the study	
☒	☐	ChIP-seq
☒	☐	Flow cytometry
☒	☐	MRI-based neuroimaging

Animals and other organisms

Policy information about studies involving animals; ARRIVE guidelines recommended for reporting animal research

Laboratory animals	Twelve 3mo old C57Bl/6J WT mice (6 males and 6 females)
Wild animals	N/A
Field-collected samples	N/A
Ethics oversight	Approved by the University of Pittsburgh IACUC

Note that full information on the approval of the study protocol must also be provided in the manuscript.

Human research participants

Policy information about studies involving human research participants

Population characteristics	The characteristics of the 106 AD and 19 cognitively normal elderly control subjects are presented in detail in Table 4 of the manuscript.
Recruitment	All subjects were participants in either the Clinical Core of the University of Pittsburgh Alzheimer Disease Research Center or the Religious Orders Study based out of the Rush University Alzheimer Disease Research Center. These sources are non-representative of the racial/ethnic diversity of the general population, and on average are more highly educated than the

	general population.
Ethics oversight	University of Pittsburgh Institutional Review Board and Committee for Oversight of Research and Clinical Training Involving Decedents

Note that full information on the approval of the study protocol must also be provided in the manuscript.

Clinical data

Policy information about clinical studies

All manuscripts should comply with the ICMJEguidelines for publication of clinical research and a completedCONSORT checklist must be included with all submissions.

Clinical trial registration	*Provide the trial registration number from ClinicalTrials.gov or an equivalent agency.*
Study protocol	*Note where the full trial protocol can be accessed OR if not available, explain why.*
Data collection	*Describe the settings and locales of data collection, noting the time periods of recruitment and data collection.*
Outcomes	*Describe how you pre-defined primary and secondary outcome measures and how you assessed these measures.*
